# Broken Ties: Black Americans’ Diminished Return of Socioeconomic Status on Subjective Well-being

**DOI:** 10.1007/s40615-025-02621-4

**Published:** 2025-09-02

**Authors:** Tiffany N. Ford, Laura D. Kubzansky, Uchechi A. Mitchell, Carol Graham

**Affiliations:** 1 Division of Community Health Sciences, School of Public Health, University of Illinois Chicago, 1603 W. Taylor St, Chicago, IL 60612 USA; 2 Department of Social and Behavioral Sciences, Harvard T.H. Chan School of Public Health, Boston, MA USA; 3 The Brookings Institution, 1775 Massachusetts Ave., NW, Washington, DC 20036 USA; 4 School of Public Policy, University of Maryland, College Park, MD 20742 USA

**Keywords:** Subjective well-being, African American, Black, Socioeconomic status, Social determinants of health, United States of America

## Abstract

Minorities’ Diminished Return (MDR) theory suggests systematically smaller effects of socioeconomic status (SES) indicators on the health of non-white populations compared to white in the United States (US). We test whether MDR theory holds with regard to subjective well-being (SWB) by investigating racial differences in the association between SES and SWB in the US using data from the Gallup Healthways survey. Our analysis included 126,669 non-Hispanic Black and white US adults. A composite SES variable comprised of financial, education, and occupation indicators was the independent variable. Life satisfaction, measured by the Cantril Ladder, was the dependent variable. Covariates included self-reported health problems, age, sex, and marital status. Race/ethnicity was tested as a moderator of the primary relationships. We estimated linear regression models in the overall sample, with and without race x SES interactions, and examined the associations between SES and SWB stratified by race/ethnicity. Findings demonstrated the strength of associations between SES and SWB differed among Black and white sub-groups. NH white adults experienced consistent increases in life satisfaction across all SES groups, while NH Black adults only begin to benefit from increasing SES when they transition from the low middle SES group to the higher SES groups. MDR theory is a relevant framework to understand potential racialized effects of SES on SWB. Multilevel policy interventions, including anti-discrimination policies, could begin to address the broken ties between SES and SWB in the US. Additional research is needed on whether MDR theory holds for other SWB domains and other SWB assets.

## Background

Subjective well-being (SWB), or self-reported quality of life, is a holistic measure used to evaluate population health. SWB predicts higher incomes, better health and longevity, and is causally linked to healthy aging [1–3], making it a topic of growing interest globally [4]. Several intergovernmental organizations, including Organization for Economic Co-operation and Development, World Health Organization, and United Nations, are encouraging countries to use SWB indicators, like life satisfaction, alongside other measures to shape their public policy decision making [5–7]. The United States (US) has begun to follow suit. Healthy People 2030, the science-backed plan that communicates the nation’s 10-year public health objectives, focused its attention on helping all people in the US to achieve their full well-being across the lifespan [8, 9]. To achieve this goal, the US aims to implement interventions and policies aimed at the physical, social, and economic environments in which people live [9]. As the number of governments incorporating SWB measures into policymaking grows, so does the need to evaluate the effectiveness of these efforts by exploring the relationships between modifiable factors, like income, education level, and occupation status, and SWB for diverse populations. An emphasis on diverse populations is necessary given noted differences in the ways that these socioeconomic factors can be utilized as a resource across groups within a nation according to race/ethnicity [10] and among Non-Hispanic Black people, specifically, in the US [11]. Such disaggregated study will encourage more inclusive growth in quality of life within populations across the world [5]. Accordingly, the present study examines race/ethnicity differences in the relationship between socioeconomic status (SES) and SWB in the US.

## The Effects of Socioeconomic Status on Subjective Well-Being

SWB is often conceptualized with three distinct domains and associated indicators [12–14]: evaluative well-being, which captures an overall assessment of ones’ life and is measured by variables like life satisfaction [13, 15, 16]; hedonic well-being, which measures positive or negative affect through point-in-time moods and emotions, like happiness, excitement, anxiety, and anger [13, 17]; and eudemonic well-being, or positive psychological functioning, which is measured in six dimensions: purpose in life, environmental mastery, autonomy, personal growth, positive relations with others, and self-acceptance [18, 19]. These different conceptualizations of SWB have different correlates [3, 5, 20]. While each domain offers useful insight into population health, limiting study to a single SWB construct allows for clear differentiation between the findings associated with each distinct conceptualization [21]. In this study, we focus on measures of SWB that capture evaluative, global assessments of life as a whole (i.e. life satisfaction) as evaluative well-being indicators tend to be more stable over time [22], are more strongly correlated with socioeconomic life conditions, such as income and employment [23–25], have been most often used in national surveys [26], and their use is recommended as targets of policy aimed at improving overall quality of life [26].

Socioeconomic status (SES) is a multidimensional construct that can be broadly understood as a social stratification system based on access to various social and economic resources [27]. While SES can be measured using objective and subjective approaches, our paper focuses on objective SES, or the material resources that an individual has [28], in order to assess the relationship between this mutable characteristic and SWB. An individual’s objective SES communicates their position within a socially constructed power hierarchy using indicators, like income, educational attainment, and employment [29, 30].

In a review of theory and literature linking SES and SWB, Kraus [31] suggests that the links between SES and SWB may depend on the extent to which people’s basic needs are met by their available resources. Income, for instance, is an important component of SES because of its ability to facilitate direct access to basic goods and services. The relationship between income and SWB has been examined extensively with income reliably positively associated with SWB in general [21], and with life satisfaction [32, 33] in particular. Educational attainment and employment status are also key measures of SES as they are understood to provide access to higher income as well as other critical resources. While higher education is less consistently predictive of higher SWB [1, 34, 35], unemployment is robustly associated with lower SWB [35, 36]. SES may be positively associated with SWB because SES resources allow people access to assets that allow them to meet their basic needs [31, 37]. By meeting those basic needs, people are overall healthier and may also have better life satisfaction.

Existing research has studied the association between SWB and the components of SES separately [27] and together in a composite SES variable [38–40]. In this study we calculate a composite SES variable to account for the multidimensional nature of SES. Treating SES as a multidimensional instead of unidimensional variable [41] allows us to gain a clearer understanding of the link between one’s placement in a social hierarchy and SWB reports. Because the individual components of SES shape one another, measuring them separately may miss their collective influence on SWB. For example, people with higher levels of education earn more money and have lower levels of unemployment according to national data [42]. Other studies that explored objective SES using composite variables were conducted in China and Turkey and found that composite SES had a direct positive relationship with SWB [38–40]. However, because none of these studies were conducted in the US their findings may not be directly generalizable to a US context.

## The Influence of Race on the Relationship Between Socioeconomic Status and Subjective Well-Being

Many studies have concluded that Black adults derive fewer benefits from higher SES attainment than white peers in the US [43–46]. For example, using longitudinal data, Shuey and Wilson [47] showed that not only do similar levels of educational attainment fail to result in the same health advantages for Black relative to white adults, but that the racial health disparity grows larger at higher levels of education, a result consistent with previous findings [45, 46]. Other research has shown that Black adults who reside in high SES neighborhoods experience increased physiological stress relative to Black adults in low SES neighborhoods, a finding that goes against the naïve hypothesis that higher SES would be associated with lower stress [44]. Indeed, nonpoor Black adults have a greater probability of worse physiological health outcomes than white people living near or below the US poverty threshold [48]. Although links between SES and SWB suggest that high SES is associated with high SWB [31], this relationship may not be consistent for all individuals within a population because higher SES does not manifest in equal outcomes across racialized groups [49]. Specifically, racism-related stressors and discrimination suppress much of the potential benefit of upward mobility for Black adults in the US [44, 50]. According to Louie and Wu [51], SES may be more protective for white versus Black adults in the US because white people may be better able to take advantage of their SES resources. This assertion aligns with the Minority Diminished Returns (MDR) theory which posits that due to the social structure in the US – a structure in which anti-Black structural racism and discrimination stratifies resources and opportunities according to race/ethnicity [52, 53] – SES resources systematically generate larger gains for white Americans than for Black Americans [54].

Support for MDR theory has been shown in a multitude of studies, whereby Black adults experienced fewer gains at higher levels of SES relative to White counterparts [43–47, 55, 56]. However, with two exceptions, no studies have evaluated this theory in relation to race differences in the benefits of SES for SWB. Assari [57] found that while high educational attainment increased happiness overall, the effect was disproportionately larger for White relative to Black adults [57]. Similarly, Assari and colleagues [58] found that increased income is associated with greater positive affect for white, but not Black, adults in the US. Across studies, Black adults with high SES measured by single indicators, like education and income, fail to obtain the levels of SWB as might be expected given their SES. Taken together, the existing literature suggests that anti-Black structural racism negatively impacts Black adults’ ability to translate their SES into resources to meet their basic needs. However, none of the previous studies explored race differences in the effect of cumulative measures of SES on SWB.

## The Current Study

Our study focuses intentionally on the US, a country where racial discrimination and systematic exclusion from socioeconomic resources according to race are/were legal (see [59] and [60] for examples of racial discrimination in US law). Due to generations of racial exclusion, SES indicators are strongly patterned by race/ethnicity in the US [61] and NH Black Americans in higher SES groups still have worse health outcomes relative to NH white Americans [46, 48]. Furthermore, between 1972 and 2016 there was a growing divide in happiness (i.e. SWB) in the US according to SES, with trends that differ for NH Black and white adults [62]. Twenge and Cooper [62] showed that while the happiness of low SES NH white adults declined over time, low SES NH Black adults’ happiness showed little change; the happiness of high SES NH white adults showed little change over time, yet the happiness of high SES NH Black adults rose. The relationships between socioeconomic factors, SWB, and race/ethnicity are complex, especially in the US. For these reasons, the US provides a unique context for examining the relationships between SES and SWB within a racialized nation.

Socioeconomic life conditions and SWB are theoretically and empirically linked [1, 34, 63, 64]. Evaluative SWB, measured in the present study by life satisfaction, is most strongly associated with SES resources (i.e. income, education, occupation) [23–25]. While the components of SES operate independent of one another, they are interrelated [65] and disadvantage in one domain of SES can shape experiences in another [47, 66]. Because the variables that make up SES are interrelated and capture different aspects of the larger SES concept [65], a composite SES indicator should be used to measure the total effect of an individual’s socioeconomic positioning on SWB [67]. SES is also distributed according to race and ethnicity within populations [49], but there is limited research examining how race/ethnicity might alter the relationship between SES and SWB [68]. Accordingly, the current study evaluates racial and ethnic differences in the association between SES and SWB among non-Hispanic (NH) Black and white adults in the US using a composite SES indicator. We collectively examine the effects of financial, educational, and occupation indicators to provide a more complete representation of an individual’s multidimensional socioeconomic positioning and its effect on SWB, allowing us to ascertain whether the benefits of SES are equally shared across racialized groups within the US population. In line with MDR theory, we hypothesize that weaker protective effects of SES on SWB will be observed for NH Black adults relative to NH white adults.

## Methods

### Sample and Design

The Gallup Daily U.S. Poll (Gallup Daily) was a nationally representative telephone survey focused on the health and SWB of US adults (≥ 18 years old) in all 50 U.S. states and the District of Columbia that ran from 2008 through 2017 [69]. We used 2016 data, which included 177,192 adults.^1^ In 2016 Gallup Daily was fielded to 500 people daily for 350 days [69]. The final sample was weighted to compensate for unequal selection probability, nonresponse, post-stratification, and to match the US population according to race/ethnicity, gender, age, education, region, population density, and phone status (i.e., cellphone only, landline only, both, and cellphone mostly) [69]. Because our research question focused on race/ethnicity differences in the relationship between SES and SWB among NH Black and white adults, we restrict the sample to NH Black and white individuals aged 18 and older (N= 147,315; 83.1%). We assessed all variables for missing data (range of missingness across all variables was between 0 and 10%), and listwise deletion was performed (n=20,646; 11.7%). The final analytic sample consisted of 126,669 adults who were either NH Black (N=13,661) or NH white (N=113,008). Relative to the analytic sample, the excluded group had a higher proportion of NH Black respondents, higher life satisfaction, and higher SES.

### Measures

We defined our outcome, life satisfaction, using the Self-Anchoring Striving Scale, or the Cantril Ladder [70] question, a widely used and validated measure of life satisfaction [71–73]. The Cantril Ladder asks participants the following prompt: “Please imagine a ladder with steps numbered from zero at the bottom to ten at the top. The top of the ladder represents the best possible life for you and the bottom of the ladder represents the worst possible life for you. On which step of the ladder would you say you personally feel you stand at this time?” The response option is an 11-point scale ranging from 0 (worst possible life) to 10 (best possible life).

Socioeconomic status (SES) was assessed by combining measures of economic standing, education level, and employment status. Like others [74, 75], we calculated the sum of scores on the components of SES. Each component was coded as a binary variable. Economic = 1 if the respondent had a household income above the median household income in 2016 ($59,039) [76] and did not report a lack of money for food or healthcare in the last year. Education = 1 if the respondent had a college or post-graduate degree. Employment = 1 if the respondent was employed part-time, self-employed, or employed full-time. The composite SES variable was then categorized to distinguish four groups: lowest SES, low middle SES, high middle SES, highest SES [reference group].

Race/ethnicity was self-reported by participants. We compared NH Black to NH white respondents using a dichotomous variable (NH white = 0 [reference group], NH Black =1).

Self-reported health, age, sex, and marital status were covariates in this analysis because each of them is related to SES and life satisfaction [39, 77–79]. Self-reported health problems =1 if the survey respondent answered yes to “Do you have any health problems that prevent you from doing any of the things people your age normally can do?” Age was measured in categories (18-24 years [reference], 25-34 years, 35-44 years, 45-54 years, 55-64 years, and 65 years and older). Females were compared to males (female=1, male=0 [reference]). There were four categories for marital status – single [reference], divorced, widowed, and married. We additionally controlled for the state of residence of the respondent as dummy variables [16] to account for potential geographic differences in SWB reports [80].

### Statistical Analyses

We conducted t-tests (for continuous variables) and chi-squared tests (for categorical variables) to assess race/ethnicity differences in sample characteristics. Ordinary least squared regression models were used to examine the associations between race/ethnicity, SES, and SWB by first treating race/ethnicity and SES category as predictor variables in the same model. Then, we treated race/ethnicity as the moderator by including interaction terms of race/ethnicity and SES. We used the predictions to graph the estimated means of SWB for NH Black and white adults at each level of SES and calculated t-tests to assess mean differences. Lastly, we estimated the associations of SES with SWB in groups stratified by race/ethnicity. Omnibus F-tests were used to test whether the additional covariates made significant improvements in our models. All models controlled for the stated covariates and used survey weights to account for the sample design. Analyses were conducted in Stata, version 18.5.

## Results

Table 1 presents characteristics of the sample by race/ethnicity; significant differences exist between NH Black and white adults for all variables except the 45-54 year old age group. NH Black adults reported lower life satisfaction relative to white adults. NH Black adults scored lower than white adults on all three components of the composite SES measure (economic, educational, and employment) and had a lower proportion of the population in the high middle and highest composite SES categories. The presence of health problems appears relatively similar among NH Black and white adults. A higher proportion of NH Black adults in the sample are < 44 years of age (Black: 54.1%; white: 39.1%) and female (Black: 55.7%; white 49.9%); fewer are married (Black: 38,1%; white: 62.4%), and more are single (Black: 42.6%; white: 20.1%) or divorced (Black: 14.8%; white: 11.3%).

**Table 1:** Descriptive statistics according to race/ethnicity (*N*=126,669)

	NH Black (*N*=13,661)	NH White (*N*=113,008)	
Variables	Mean (SE) or Counts (%)	Mean (SE) or Counts (%)	*p* Value
Life satisfaction (0-10)	6.80 (2.12)	7.03 (1.90)	<0.001
Composite socioeconomic status (0-3)			<0.001
Lowest SES	4,540 (33.2%)	25,501 (22.6%)	
Low middle SES	4,623 (33.8%)	32,116 (28.4%)	
High middle SES	2,714 (19.9%)	32,728 (29.0%)	
Highest SES	1,784 (13.1%)	22,663 (20.1%)	
Economic component of SES (0,1)	0.31 (0.46)	0.52 (0.50)	<0.001
Education component of SES (0,1)	0.26 (0.44)	0.36 (0.48)	<0.001
Employment component of SES (0,1)	0.56 (0.50)	0.59 (0.49)	<0.001
Health problems (0,1)	0.21 (0.41)	0.23 (0.42)	<0.001
Age (in categories)			
18-24 years	2,158 (15.8%)	10,969 (9.7%)	<0.001
25-34 years	2,731 (20.0%)	16,311 (14.4%)	<0.001
35-44 years	2,497 (18.3%)	17,004 (15.0%)	<0.001
45-54 years	2,536 (18.6%)	20,577 (18.2%)	0.237
55-64 years	2,169 (15.9%)	22,933 (20.3%)	<0.001
65+ years	1,571 (11.5%)	25,214 (22.3%)	<0.001
Sex			
Male	6,048 (44.3%)	56,637 (50.1%)	<0.001
Female	7,613 (55.7%)	56,371 (49.9%)	<0.001
Marital status			
Single	5,818 (42.6%)	22,687 (20.1%)	<0.001
Divorced	2,015 (14.8%)	12,807 (11.3%)	<0.001
Widowed	623 (4.6%)	7,022 (6.2%)	<0.001
Married	5,205 (38.1%)	70,493 (62.4%)	<0.001

*SE* Standard error, *SES* Composite Socioeconomic Status; made up of three SES components: Economic (1=Household income above median and not lacking money for food or healthcare in past year), Education (1=College graduate or post-graduate), Employment (1=Employed part-time, full-time, or self-employed)

Data comes from Gallup Daily 2016 survey year

**Fig. 1:**
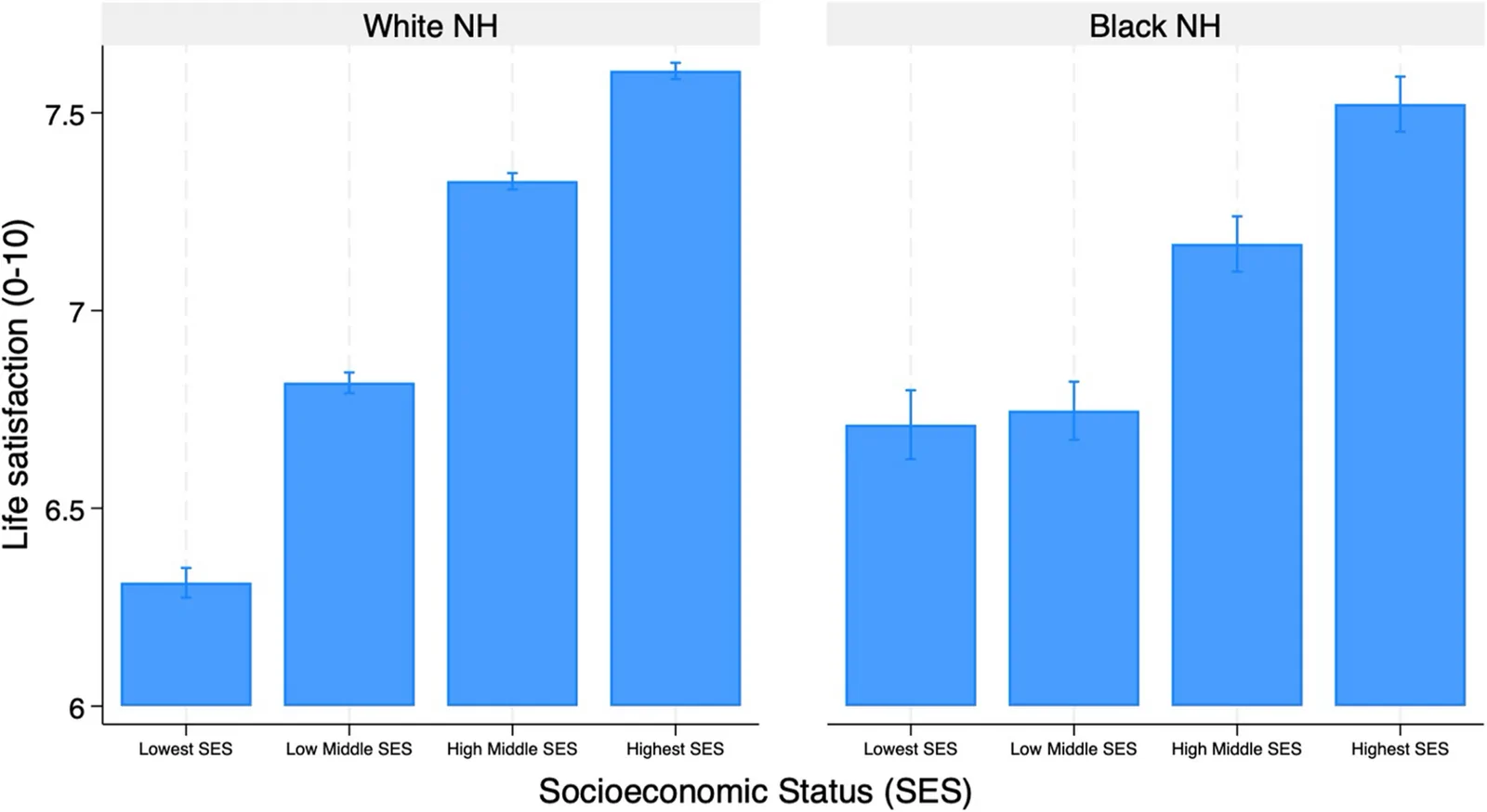
The association between socioeconomic status and life satisfaction by race/ethnicity: Gallup daily, 2016 (*N*=126,669)

Table 2 shows the main effect of race/ethnicity on life satisfaction and its interaction with SES. After adjusting for all covariates, NH Black adults reported slightly higher levels of life satisfaction relative to NH white people (Model 1: β = 0.06). Individuals with lower SES had significantly lower life satisfaction compared to the highest SES group (Model 1: β = −1.20). This difference in life satisfaction decreased in a dose-response manner at higher levels of SES. Results from Model 2 including interaction terms for race/ethnicity x SES show a statistically significant Black-white difference in life satisfaction among the lowest SES group (β = 0.48, *p*<0.001), but the individual interaction terms for the other SES groups were not significant. Lowest SES NH Black adults had significantly higher SWB than lowest SES NH white adults (Figure 1). Within race/ethnicity, both NH Black and white individuals with higher versus lower SES had significantly higher levels of life satisfaction. NH white adults experienced consistent increases in life satisfaction across all SES groups, while NH Black adults only begin to benefit from increasing SES when they transition from the low middle SES group to the higher SES groups.

**Table 2:** Racial/ethnic differences in the association between composite socioeconomic status and life satisfaction in the US: Gallup Daily, 2016 (*N*=126,669)

	Model 1		Model 2	
Variables	β	SE	β	SE
Interaction effects				
Black NH x Lowest SES ^a^			0.48***	**(0.06)**
Black NH x Low Middle SES ^a^			0.01	(0.05)
Black NH x High Middle SES ^a^			−0.07	(0.05)
Main effects				
Black NH ^b^	0.06*	(0.02)	−0.08*	(0.04)
Lowest SES ^c^	−1.20***	(0.02)	−1.29***	(0.02)
Low Middle SES ^c^	−0.80***	(0.02)	−0.79***	(0.02)
High Middle SES ^c^	−0.29***	(0.01)	−0.28***	(0.01)
Covariates				
Health problems ^d^	−0.76***	(0.02)	−0.76***	(0.02)
25-34 years ^e^	−0.21***	(0.03)	−0.21***	(0.03)
35-44 years ^e^	−0.27***	(0.03)	−0.27***	(0.03)
45-54 years ^e^	−0.25***	(0.03)	−0.25***	(0.03)
55-64 years ^e^	−0.03	(0.03)	−0.03	(0.03)
65+ years ^e^	0.56***	(0.03)	0.57***	(0.03)
Female ^f^	0.29***	(0.01)	0.29***	(0.01)
Married ^g^	0.40***	(0.02)	0.40***	(0.02)
Divorced/separated ^g^	−0.14***	(0.03)	−0.13***	(0.03)
Widowed ^g^	0.12***	(0.03)	0.13***	(0.03)
Constant	7.63***	(0.07)	7.64***	(0.07)
Model fit statistics				
R-squared	0.129		0.131	
Omnibus *F*-test			32.33	
Prob>*F*			0.00	

The bold values show significance of the interaction effects of race/ethnicity and socioeconomic status

State controls are included but not displayed

*SE* Standard error, *SES* Composite Socioeconomic Status; made up of three SES components: Economic (1=Household income above median and not lacking money for food or healthcare in past year), Education (1=College graduate or post-graduate), Employment (1=Employed part-time full-time or self-employed)

^a^NH Black in highest SES category, ^b^NH white, ^c^highest SES category, ^d^no health problems, ^e^18-24 years, ^f^male, ^g^single

* *p*<0.05 ** *p*<0.01 *** *p*<0.001

In Table 3 we further explore the associations between SES category and life satisfaction in race-stratified models. Among NH Black individuals, Model 1 includes the covariates; Model 2, adds SES. We do the same for NH white individuals in Model 3 and Model 4. The addition of the SES composite measure as a covariate increased the explanatory power of the models for both NH Black and NH white adults; however, this change was not consistent across race/ethnicity, suggesting that the associations between SES and SWB are weaker among NH Black versus white adults in the US.

**Table 3:** The association between composite socioeconomic status and life satisfaction in the US according to race/ethnicity: Gallup Daily, 2016

	Black NH (*n*= 13,661)				White NH (*n*=113,008)			
	Model 1		Model 2		Model 3		Model 4	
Variables	β	SE	β	SE	β	SE	β	SE
Lowest SES ^a^			−0.89***	(0.06)			−1.28***	(0.02)
Low Middle SES ^a^			−0.78***	(0.05)			−0.79***	(0.02)
High Middle SES ^a^			−0.36***	(0.05)			−0.28***	(0.01)
Health problems	−0.83***	(0.06)	−0.66***	(0.06)	−1.06***	(0.02)	−0.78***	(0.02)
Female	0.21***	(0.04)	0.20***	(0.04)	0.23***	(0.01)	0.31***	(0.01)
25-34 years	0.17*	(0.08)	0.08	(0.08)	−0.16***	(0.03)	−0.31***	(0.03)
35-44 years	0.16*	(0.08)	0.02	(0.08)	−0.18***	(0.03)	−0.38***	(0.03)
45-54 years	0.46***	(0.08)	0.33***	(0.08)	−0.25***	(0.03)	−0.41***	(0.03)
55-64 years	0.63***	(0.09)	0.56***	(0.09)	−0.10**	(0.03)	−0.19***	(0.03)
65+ years	0.92***	(0.09)	0.93***	(0.09)	0.32***	(0.03)	0.44***	(0.03)
Married	0.33***	(0.05)	0.18***	(0.05)	0.63***	(0.02)	0.47***	(0.02)
Divorced/separated	−0.17*	(0.07)	−0.19**	(0.07)	−0.16***	(0.03)	−0.10***	(0.03)
Widowed	0.05	(0.12)	0.06	(0.12)	0.07	(0.04)	0.18***	(0.04)
Constant	6.63***	(0.18)	7.42***	(0.19)	6.94***	(0.08)	7.68***	(0.08)
Model fit statistics								
*F* value	10.68		15.92		113.33		169.19	
Prob>*F*	0.00		0.00		0.00		0.00	
R-squared	0.055		0.072		0.102		0.146	
Δ in R-squared			0.018				0.045	
Omnibus* F*-test			89.74				1243.77	
Prob>*F*			0.00				0.00	

State controls are included but not displayed

*SE* Standard error, *SES* Composite Socioeconomic Status

^a^highest SES category

*p<0.05 **p<0.01 ***p<0.001

## Discussion

This study explored the association between SES and evaluative SWB for Black and white adults in the US. We have three main findings to report. First, we found that as SES increases, life satisfaction also increases, but the strength of the SES and SWB association differs between NH Black and white adults at the lowest level of SES. We found a significant positive interaction between race/ethnicity and the lowest SES category, indicating that being low SES is associated with less reduction in life satisfaction for NH Black versus white individuals. Additionally, the SWB reports of NH Black adults in the lowest SES category were significantly higher than those of lowest SES white adults. Second, the interactions between race/ethnicity and the higher SES categories (i.e. low middle, high middle) were not significant, suggesting that the SES-SWB relationship that was evident at the lowest level of SES for NH Black adults was no longer present at higher levels of SES. Third, we found that the addition of SES in our model better explained life satisfaction for NH white adults in the US than it did NH Black adults. Our findings provide some support for MDR theory which seeks to explain observed smaller health gains from SES indicators for Black relative to white adults. While low SES is associated with lower SWB among both Black and white adults, the association is less potent among Black adults. These findings suggest that the ties between SES and SWB are at least partially broken in parts of US society.

The leading explanation for the higher SWB of lowest SES Black adults is greater resilience, or the ability to maintain health “in spite of a range of psychosocial risk factors” among some minoritized groups [15, 16]. Assari [54] suggests the development of resilience is systemic and Wadsworth and Pendergast [81] argue that a history of oppression, prejudice, and discrimination for Black Americans may have led to higher levels of resilience in response to times of challenge among the racialized group. However, important to note, is that our understanding of how resilience manifests and its downstream effects is nascent. For example, resilience may reflect individual actions, such as active coping, which may not necessarily be protective across all outcomes. Active coping has been described as “the process of taking active steps to attempt to get rid of the stressor or to reorganize its effects” [82]. Active coping with anti-Black racism, specifically, may mean that individuals are speaking up about unfair treatment, working harder to try to change the situation or to prove others wrong [83]. Wiley and co-authors [84] attribute Black people’s high levels of self-reported health and higher mental health levels relative to white people to actively and excessively coping with stress, which is then interpreted as resilience. While the higher SWB of lowest SES Black relative to white adults may be reflective of individual-level resilience, this explanation does not hold at higher SES levels.

Our examination of race/ethnicity difference in the effects of SES on the level of SWB in the higher SES categories did not reveal statistically significant results. A potential explanation for failing to find significant interactions between race/ethnicity and the higher SES categories is a threshold effect. We found that among NH Black adults, being lowest SES is associated with less reduction in life satisfaction relative to white adults; however, once both NH Black and white individuals reach low middle SES, it seems that the benefits of higher SES are similar across race/ethnicity. This finding begs the question: what is it about the transition from lowest SES to low middle SES that leads to race/ethnicity differences in the SES-SWB relationship? And still, why are these same mechanisms not operating at higher levels of SES? Life satisfaction reports among NH Black adults in the lowest SES category were significantly higher than those of lowest SES white adults; NH Black adults in the high middle and highest SES categories had significantly lower life satisfaction relative to NH white adults in the same SES categories.

Perhaps higher SES Black adults do not experience the same effect as lowest SES Black adults because of the difficulties that that they experience when trying to translate their SES resources into SWB resources. Previous research on the reduced health returns of education [56] and income [85] suggest that, due to anti-Black structural racism in social and economic life, Black adults have a harder time using their SES resources. For example, Black people have a more difficult time translating higher education into higher income [79] due to labor market discrimination in hiring [86] and racialized and gendered unemployment and wage gaps [87–89]. There is an extra cost to upward social mobility for Black people [54], which may manifest as no additional gain from higher SES for the SWB of Black relative to white adults above and beyond the main effect. Given that SES better explained white life satisfaction than it did Black, perhaps there are other modifiable factors that make a stronger contribution to the life satisfaction of higher SES Black adults in the US.

This study has several strengths. First, it is among the few to evaluate MDR theory in relation to race/ethnicity differences in SES and SWB and one of the few studies to use a multidimensional measure of SES to examine MDR theory in the US. By integrating financial, educational, and employment indicators into a single measure, this study provides a novel perspective on the comprehensive role that an individual’s socioeconomic positioning plays in shaping SWB. Our multidimensional examination of SES and its association with SWB sheds light on the ways that SWB resources may translate into different quality of life outcomes across racialized groups within a nation. Second, this study benefits from the use of Gallup Daily, a very large nationally representative dataset that facilitates identifying robust associations between the key variables across sub-groups of the population. Third, this study helps to fill a gap in the evidence on how race/racism shapes SWB in the US [90]. However, this study has limitations as well. First, our study design was cross-sectional and used 2016 data. Further quantitative analysis using longitudinal datasets to study racial/ethnic differences in the associations between the SWB resources and SWB over time will be better equipped to explore causality. Additionally, future research using more recent data can offer a perspective on how the societal shifts since 2016 (i.e. because of COVID-19, the 2020 “racial reckoning” [91], both Trump administrations) may impact the relationships explored. Second, this study uses the Cantril Ladder question to measure SWB. While the Cantril Ladder is a valid measure of SWB, it has not been validated among Black respondents, specifically. The work of validating this commonly used SWB measure among Black populations remains an open task for future researchers. Third, this analysis compares the associations between SES and SWB for NH Black and white adults in the US using NH white adults as the reference group. Given the heterogeneity that exists within race/ethnicity, future research should consider exploring the associations between SES – and other SWB resources – in within group analyses.

## Conclusions

The results of this study suggest that the protective effects of increasing social resources as marked by SES on SWB differ depending on race/ethnicity. Racialized associations between the resources that contribute to good quality of life, like SES, and SWB likely reflect differential returns on well-being resources for Black people in the US. The higher SWB levels among lowest SES Black adults may reflect active coping and individual-level resilience, while the SWB level of higher SES Black Americans is possibly a reflection of anti-Black structural racism in US society, which blocks Black adults from realizing the full benefits of their SES and from translating that SES into expected outcomes, like higher SWB. Our findings demonstrate that race and racism are essential components to consider when studying SWB and suggest that resilience is an incomplete explanation of these racialized findings. Indeed, authors who write from a Black queer and trans perspective have problematized the concept of resilience, arguing that we should consider “resilience as scar tissue” and suggesting resilience is actually a “marker of health inequities” [92]. While our findings provide some support for MDR theory, they also suggest that there is much more to learn about the resilience of racial/ethnic minorities in the US and whether such resilience might have other costs.

A multilevel policy response is needed to address the circumstances in which the ties between SES and SWB appear to be broken in US society [54]. First, identifying and avoiding policies which diminish Black adults’ return on SES resources is essential. Second, federal, state, and local legislation is necessary to reduce interpersonal and structural discrimination. These policies must be implemented and adequately enforced if they are meant to address the broken ties between SES and SWB in the US. Future analysis should focus on the associations between SWB and other SWB-promoting resources for Black Americans and other non-white groups that experience systematic discrimination in the US. Focusing policy attention and further research on these potentially racialized associations may help to target investments in SWB-promoting resources in ways that will yield maximum SWB benefit for Black Americans and other non-white groups. This focus may also shed necessary light on all that is still unknown about how race/racism shapes relationships between the resources that it takes to be well and quality of life in the US.

## Data Availability

The data presented in this article cannot be made readily available by the authors because the data must be accessed by subscribing to Gallup Analytics. Requests to access the data should be directed to Gallup Analytics at https://www.gallup.com/analytics/213617/gallup-analytics.aspx.

## Footnotes

1 Gallup Daily is a proprietary dataset that we have permission to use because the last author (CG) is a Senior Scientist with Gallup Inc. 2016 is the latest year of Gallup Daily data that the first author (TF) has access to.
